# First record of the genus *Trispinaria* Quicke, 1986 (Hymenoptera, Braconidae, Braconinae) in Vietnam, with descriptions of two new species

**DOI:** 10.3897/zookeys.996.56562

**Published:** 2020-11-24

**Authors:** Nguyen Thi Oanh, Khuat Dang Long, Pham Quynh Mai, Nguyen Van Dzuong

**Affiliations:** 1 Dong Thap University, Cao Lanh City, Dong Thap, Vietnam Dong Thap University Cao Lanh Vietnam; 2 Institute of Ecology & Biological Resources, Vietnam Academy of Science & Technology, 18 Hoang Quoc Viet Road, Cau Giay, Ha Noi, Vietnam Institute of Ecology & Biological Resources, Vietnam Academy of Science & Technology Ha Noi Vietnam; 3 Tay Bac University, Son La City, Vietnam Tay Bac University Son La Vietnam

**Keywords:** Australasian region, Ichneumonoidea, new record, Oriental region, parasitoid, taxonomy, wasp

## Abstract

Two new species of the genus *Trispinaria* Quicke, 1986, from Vietnam, viz. *T. seminigra* Long, **sp. nov.** and *T. vietnamica* Long, **sp. nov.**, are described and fully illustrated. Additionally, this is the first record of the genus *Trispinaria* in Vietnam. A checklist with distributions of previously described species of the genus *Trispinaria* is given. Comparative characters of the Vietnamese species are provided and modified key couplets are provided to facilitate their identification.

## Introduction

*Trispinaria* was described by Quicke (1986) from SW Sulawesi, including only the type-species *Trispinaria priscicolorus* Quicke,1986. *Trispinaria* is an aberrant and rather uniform genus of the subfamily Braconinae. *Trispinaria* species occur over the Oriental and Australasian regions; van Achterberg (1991) listed and keyed the eight species. Subsequently, Wang et al. (2003) described one new species from China, resulting in eight species described from the Oriental region. A ninth species is recorded from the Australasian region.

Quicke (1988) placed *Trispinaria* as sister group of *Physaraia* Shenefelt, 1978, because of the fused first and second metasomal tergites and propodeal sculpture. However, van Achterberg (1991) reported that more likely the genus is closely related to the Oriental genus *Pseudospinaria* Enderlein, 1920. Van Achterberg based his conclusion on the following suit of characters: long and curved vein 1r-m of the hind wing; protruding median carina of metanotum; united dorsal carinae of the first tergite; pair of converging grooves of the second tergite, the third-fifth metasomal tergites possess spines; the first subdiscal cell of the fore wing is more slender; the second tergite has a pair of converging grooves; and the propleuron is concave ventrally. *Pseudospinaria* differs from *Trispinaria* by having both basal segments of the metasoma movably jointed, a large fore wing second submarginal cell, bifurcate tarsal claws, and reduced scutellar sulcus. A detailed diagnosis of the genus *Trispinaria* was given by van Achterberg (1991).

The biology of *Trispinaria* is unknown but based on the united and heavily sclerotised basal metasomal tergites, van Achterberg (1991) suggested the ovipositor could insert into a hard substrate. Following the points of van Achterberg (1991), the colouration of the wasps corroborates the idea that they occur in open, sunny, and dry types of forest. In tropical rain forests most of the large braconid wasps possess a dark reddish brown and black colour pattern.

## Materials and methods

The specimens studied, including holotypes and some paratypes, are housed in the Institute of Ecology & Biological Resources (**IEBR**) at Ha Noi; other paratypes have been donated to and are deposited in the American Museum of Natural History (**AMNH**), New York, USA, and the Vietnam National Museum of Nature (**VNMN**), Ha Noi, Vietnam.

### Morphology

For terminology used in this paper, see van Achterberg (1993), sculpture terms are based on Harris (1979), and vein terminology follows the modified Comstock-Needham system (van Achterberg 1993). For a key to the Old World genera of the subfamily Braconinae, see Quicke (1987).

We used an Olympus SZ61 binocular microscope together with fluorescent lamps for sorting, identification and descriptions. The key to species and the descriptions of species are based on females. Measurements are taken under an Olympus SZ40 binocular microscope. The scale-lines of the plates (habitus and fore wing) represent 1.0 mm. The photographs were made with a Sony 5000 digital camera attached to a Nikon SMZ 800N binocular microscope connected to a PC at IEBR and processed with Adobe Photoshop CS5 to adjust the size and background. A distribution map of two new species of *Trispinaria* was made using Paraview (https://paraview.org).

Abbreviations used in this paper are as follows:

**POL** minimum postocellar line;

**OOL** minimum ocular-ocellar line;

**OD** maximum diameter of posterior ocellus;

‘**Bracn. + number**’ code number indexing for Braconinae specimens in the collection at IEBR and VNMN;

**MT** Malaise trap;

**N** north;

**NC** north central;

**NE** northeastern;

**NP** National Park;

**NW** northwestern;

**S** south.

Institutional abbreviations are as follows:

**AMNH** American Museum of Natural History, New York, USA;

**IEBR** Institute of Ecology & Biological Resources, Vietnam Academy of Science and Technology, Ha Noi, Vietnam;

**STCT** Department of Insect Ecology at IEBR;

**VNMN** Vietnam National Museum of Nature, Vietnam Academy of Science and Technology, Ha Noi, Vietnam.

In Vietnam, the distribution of the species is given in order of areas and provinces from north to south, and outside Vietnam, distribution of species follows an alphabetical order.

## Results

**Class Hexapoda Blainville, 1816**

**Order Hymenoptera Linnaeus, 1758**

**Superfamily Ichneumonoidea Latreille, 1802**

**Family Braconidae Nees, 1811**

**Subfamily Braconinae Nees, 1811**

**Tribe Braconini Nees, 1811**

### Genus Trispinaria

Taxon classificationAnimaliaHymenopteraBraconidae

Quicke, 1986

0EA8CCA2-6522-5F4A-B66C-985B45460ED4

Trispinaria Quicke, 1986a: 10 & 1987: 134.

#### Type-species.

*Trispinaria priscicolorus* Quicke, 1986 (monobasic and original designation).

#### Checklist and distribution of *Trispinaria* species.

*T. albibasis* van Achterberg, 1991: figs 33, 34, 36/ Oriental: Malaysia (Peninsular).

*T. betremi* van Achterberg, 1991: figs 31, 32, 35/ Oriental: Indonesia (Java).

*T. chinensis* Wang, Chen & He, 2003: figs 1–9/ Oriental: China (Guangxi).

*T. maculata* van Achterberg, 1991: figs 41–44/ Oriental: China-Taiwan; India; Malaysia (Peninsular); Singapore; Sri Lanka.

*T. priscicolorus* Quicke, 1986a: figs 1–11/ Australasian: Indonesia (Sulawesi).

*T. sannio* (Enderlein, 1920): figs 37–40/ Oriental: Indonesia; Singapore

*T. setosa* van Achterberg, 1991: figs 26–30/ Oriental: Indonesia (Bali)

*T. seminigra* Long, sp. nov./ Oriental: NE Vietnam (Tuyen Quang), N Vietnam (Ninh Binh), NC Vietnam (Ha Tinh).

*T. sulcata* van Achterberg, 1991: figs 22–25/ Oriental: Philippines (Mindanao, Mindoro).

*T. unicolor* van Achterberg, 1991: figs 17–20/ Oriental: Philippines.

*T. vietnamica* Long, sp. nov./ Oriental: N Vietnam (Thai Nguyen), NW Vietnam (Son La, Hoa Binh), S Vietnam (Pleicu).

### Trispinaria seminigra

Taxon classificationAnimaliaHymenopteraBraconidae

Long sp. nov.

5DF0F293-205C-587D-8640-7BC4F1386D24

http://zoobank.org/C6F2CE0E-C33C-48E8-95D2-F2BE2D120B7D

Figs 1 , 2–12

#### Material.

***Holotype***, ♀, “Bracn.1503” (IEBR), NE Vietnam: Tuyen Quang, Na Hang NP, Son Phu, forest, MT, 22°17'34"N, 105°28'19"E, 561 m, 15.iv.2018, KD Long. ***Paratypes***, 3 ♀, “Bracn.**768**” (IEBR), N Vietnam: Ninh Binh, Cuc Phuong NP, forest, 20°19'N, 105°35'E, 180 m, sweeping, 9.v.2002, KD Long, “Bracn.1411” (VNMN), NE Vietnam: Tuyen Quang, Na Hang, Thanh Tuong, forest, MT, 22°19'01"N, 105°24'02"E, 162 m, 5.xi.2016, KD Long; “Bracn.**710**” (AMNH), NC Vietnam: Ha Tinh, Huong Son, forest, 18°13'N, 105°24'E, 900 m, 20–28.iv.1998, AMNH, K. Long.

#### Description.

**Figure 1.**
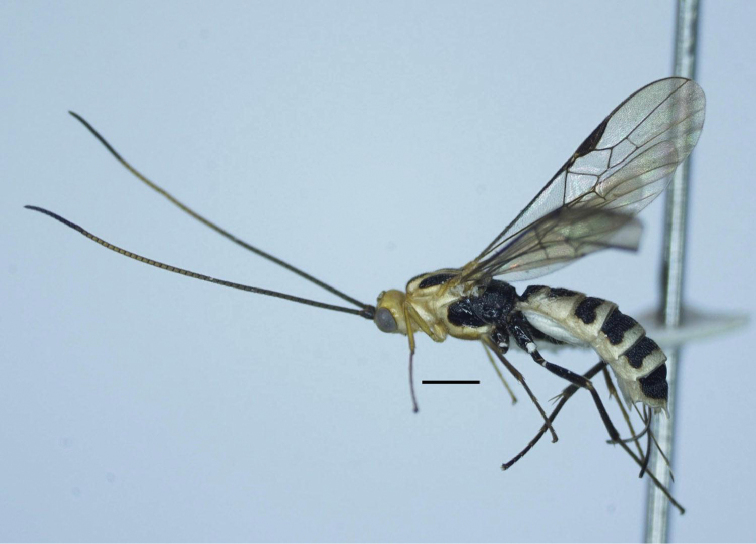
*Trispinaria seminigra* Long, sp. nov., holotype, female, habitus, lateral view.

Holotype, female, body length 6.2 mm, fore wing length 5.7 mm, antenna 7.3 mm, ovipositor sheath 1.5 mm (Fig. 1).

**Figures 2–12.**
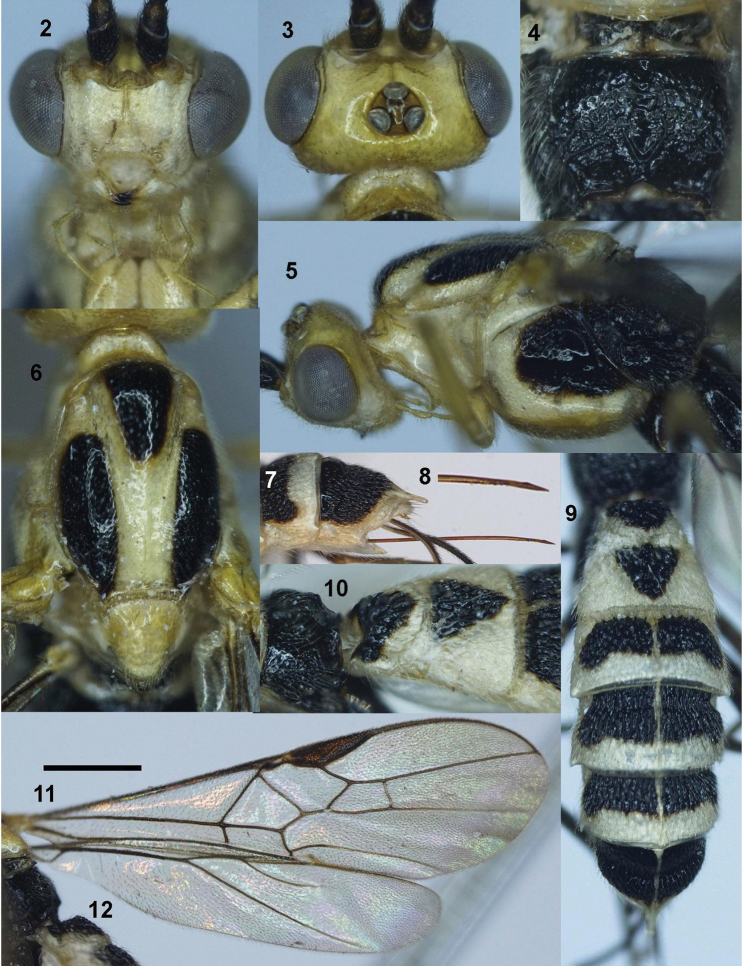
*Trispinaria seminigra* Long, sp. nov., holotype, female. **2** head, frontal view **3** head, dorsal view **4** propodeum **5** mesopleuron **6** mesonotum **7** fifth and sixth metasomal tergites, lateral view **8** apex of ovipositor **9** metasoma **10** first and second metasomal tergites, lateral view **11** fore wing **12** hind wing.

***Head***. Antenna with 58 antennomeres; length of third and fourth antennomere 1.75 (7 : 4) and 1.5× their width (6 : 4); length of subapical antennomere 1.3× its width (4 : 3); in frontal view, width of face 1.9× its length (25 : 13) (Fig. 2); length of maxillary palp 0.7× height of head (25 : 37); face flattened, transversely rugose, triangular area upper clypeus smooth (Fig. 2); malar space as long as basal width of mandible (8 : 8); clypeus convex medially, depressed laterally, its apical margin concave and with distinct carina (Fig. 2); distance between tentorial pits 1.7× distance from pit to eye margin (10 : 6); in lateral view, eye 3.0× temple (18 : 6); in dorsal view, head 1.7× as wide as long (49 : 29); in dorsal view, width of head 1.7× median length (49 : 29); eye 3.0× as long as temple (21 : 7); POL : OD : OOL = 3 : 5 : 10; eye 3.0× temple (21 : 7); frons flat, smooth, with fine median groove (Fig. 3).

***Mesosoma***. Length of mesosoma 1.65× its height (78 : 47); propleuron shallow, finely crenulate medially (Fig. 5); middle lobe of mesoscutum without impressions anteriorly; notauli deeper anteriorly, wider and flat posteriorly, almost smooth with faint median carina (Fig. 6); median lobe of mesoscutum without groove; mesoscutal lobes shiny, sparsely finely punctate; prescutellar sulcus narrow, crenulate; scutellum sparsely punctate; mesopleuron largely smooth, with large sparse punctures dorsally (Fig. 5); metapleuron punctate; propodeum with distinct V-shaped carina posteriorly (Fig. 4); median depression sparsely crenulate anteriorly, almost sooth posteriorly; lateral areola-like areas almost coriaceous.

***Wings***. Length of fore wing 3.2× its maximum width (240 : 75); length of pterostigma 3.8× its width (42 : 11); fore wing vein SR1 4.8× as long as vein 3-SR (67 : 14); r : 3-SR : SR1 = 13 : 14 : 67; cu-a interstitial, weakly inclivous (Fig. 11), cu-a : 2-CU1 = 7 : 26; 2-SR : 3-SR : r-m = 15 : 14 : 11; second submarginal cell of fore wing less robust (Fig. 11); hind wing vein 1-M weakly curved basally (Fig. 12); vein 1r-m of hind wing largely united with 1-SC+R.

***Legs***. Hind coxa sparsely setose; length of femur, tibia and basitarsus of hind leg 4.9×, 10.6× and 8.0× their width, respectively; length of hind inner and outer tibial spurs 0.5× and 0.4× hind basitarsus (16 : 32)(13 : 32), respectively; length of hind basitarsus 0.4× hind tibia (32 : 74) and 0.7× second-fifth tarsus (32 : 44); hind tarsal claw with large lobe.

***Metasoma***. Length of first tergite 0.9× its apical width (27 : 30), with basal excavation narrow and deep (Fig. 10); antero-lateral groove shallow, sparsely crenulate; median length of second tergite 0.9× third tergite (25 : 27; first metasomal tergite posteriorly, second-sixth metasomal tergites entirely coarsely reticulate (Fig. 9); tooth of sixth tergite developed (Fig. 7); latero-apical groove narrow, crenulate (Fig. 7); length of ovipositor sheath 0.26× fore wing (15 : 57); ovipositor with dorsal nodus; subapical ventral margin of ovipositor underneath nodus with serrations, but apico-ventrally without serrations (Fig. 8).

***Colour***. Head yellow; mesosoma and metasoma pale yellow; scapus brown; twenty middle antennomeres yellow; palpi and stemmaticum brownish yellow; mesoscutal lobes black, except median lobe laterally and posteriorly, lateral lobes anteriorly pale yellow; mesopleuron yellow ventrally, black dorsally; metapleuron black; scutellum pale yellow; metanotum and propodeum black; fore legs pale yellow; middle coxa, trochanter and trochantellus brown; middle femur brown, except outer side yellow; middle tibia and tarsus yellowish brown; hind legs black; hind tibial spurs pale yellow; pterostigma and veins brown; wing membrane subhyaline basally and medially, except fore wing membrane yellowish brown apically; first and second metasomal tergites black medio-basally, pale yellow laterally and apically; third-fifth metasomal tergites black basally, pale yellow apically; sixth metasomal tergite black, yellow apically; ovipositor sheath brown; ovipositor brownish yellow.

#### Variations.

Length of body 5.8–7.7 mm, of fore wing 5.3–6.7 mm; antenna with 57–65 antennomeres; 16–26 middle antennomeres yellow or antenna brown entirely; stemmaticum brownish yellow; vein SR1 of fore wing 4.0–4.9× vein 3-SR; length of ovipositor sheath 0.22–0.25× fore wing; middle coxa and tarsus brownish yellow; middle femur and tibia yellow; ovipositor yellow.

**Male**. Unknown.

#### Biology.

Unknown.

#### Etymology.

From *semi* (Latin for half) and *niger* (Latin for black), because the mesopleuron is black dorsally in contrast to the yellow ventral half.

#### Distribution.

N Vietnam: Tuyen Quang, Ninh Binh; NC Vietnam: Ha Tinh.

#### Notes.

*Trispinaria seminigra* sp. nov. differs from *T. vietnamica* sp. nov. by having: median length of first metasomal tergite 0.9× as long as its apical width; propleuron shallow, finely rugose; fore wing vein cu-a slightly postfurcal and distinctly inclivous; hind wing vein 1-M almost straight basally; middle coxa dark brown; mesopleuron black dorsally; ovipositor apico-ventrally without serrations, except pre-apical ventral margin underneath with serrations.

The new species, *T. seminigra* sp. nov., is close to *T. sannio* (Enderlein), from Indonesia and Singapore by sharing the following characters: vein 1r-m of hind wing nearly united with vein 1-SC+R; apical half of subbasal cell of fore wing largely glabrous; and frons smooth. The new species can be inserted into the key by van Achterberg (1991) as follows:

**Table:** 

7a.	Whole antenna black; face coarsely punctate, at most with some rugae dorsally; second submarginal cell of fore wing comparatively robust (cf. fig. 37 in van Achterberg 1991); vein cu-a of fore wing distinctly inclivous, more than vein 3-CU1 (cf. figs 37, 40 in van Achterberg 1991); length of ovipositor sheath 0.5× fore wing; length of vein SR1 of fore wing 3.9–4.4× vein 3-SR; metapleuron with small black patch; propodeum with pair of two large black patches. Indonesia, Singapore	***T. sannio* (Enderlein, 1920)**
a’.	Antenna dark brown basally and apically, yellowish medially; face transversely rugose, except triangular area upper clypeus smooth; second submarginal cell of fore wing slender (Fig. 11); vein cu-a of fore wing less inclivous (Fig. 11); length of ovipositor sheath 0.3× fore wing; length of vein SR1 of fore wing 4.0–4.9× vein 3-SR; mesopleuron dorsally, metapleuron and propodeum entirely black (Figs 4, 5). Vietnam	***T. seminigra* Long, sp. nov.**

### Trispinaria vietnamica

Taxon classificationAnimaliaHymenopteraBraconidae

Long sp. nov.

041873CB-24A3-5096-9FCD-8BC316A82EDE

http://zoobank.org/4F51C7EE-573E-4186-9F42-ACCC78B59BB1

Figures 13 , 14–24

#### Material.

***Holotype***, ♀, “Bracn.376” (IEBR), S Vietnam: Pleicu, Dak Do, Ha Bau, > 800 m, bushes, 08.vi.2005, KD Long. ***Paratypes***: 6 ♀, “Bracn.377” (IEBR), data as holotype; “Bracn.708” (IEBR), NE Vietnam: Thai Nguyen, Dai Tu, Cat Ne, MT, orchard, 21°31'24"N, 105°29'39"E, 302 m, 30.xi.2006, KD Long; “Bracn.708” (VNMN), ibid. but 5.xi.2006, KD Long; “Bracn.747” (IEBR), ibid, but 25.xii.2006, KD Long; “Bracn.1481” (IEBR), NW Vietnam: Son La, coffee orchard, MT, 21°18'06"N, 103°55'36"E, 663 m, 10.iii.2018, KD Long, NV Dzuong; “Bracn.1491” (IEBR), NW Vietnam: Hoa Binh, Luong Son, Thanh Lap, fruit orchard, MT, 20°48'46"N, 105°37'58"E, 20 m, 5.ii.2018, STCT.

#### Description.

**Figure 13.**
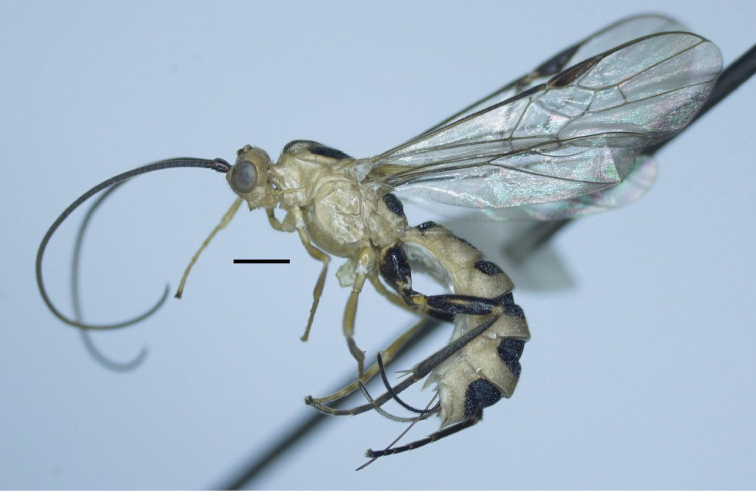
*Trispinaria vietnamica* Long, sp. nov., holotype, female, habitus, lateral view.

Holotype, female, body length 8.3 mm, fore wing length 7.2 mm, antenna 7.7 mm, ovipositor sheath 1.8 mm (Fig. 13).

**Figures 14–24.**
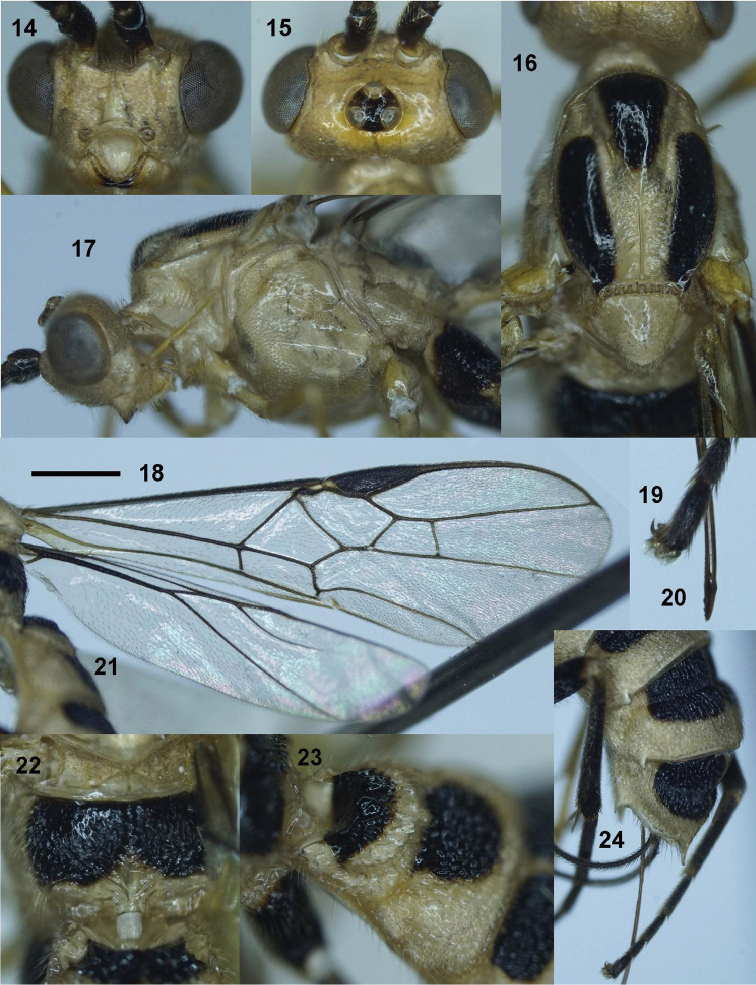
*Trispinaria vietnamica* Long, sp. nov., holotype, female **14** head, frontal view **15** head, dorsal view **16** mesonotum **17** mesopleuron **18** Fore wing **19** hind telotarsus and tarsal claw **20** apex of ovipositor **21** hind wing **22** propodeum **23** first and second metasomal tergites, lateral view **24** fifth and sixth metasomal tergites, lateral view.

***Head***. Antenna with 74 antennomeres; length of third and fourth antennomeres 1.6× and 1.4× their width, respectively (8 : 5) (7 : 5); length of subapical antennomere as long as wide (3 : 3); in frontal view, width of face 0.5× its length (33 : 16) (Fig. 14); length of maxillary palp 0.8× height of head (36 : 45); malar space 0.8× as long as basal width of mandible (10 : 13); face flattened, punctate-coriaceous and rather matt; clypeus convex medially, depressed laterally, its apical margin concave and with distinct carina (Fig. 14); distance between tentorial pits 1.6× distance from pit to eye margin (13 : 8); in lateral view, eye 2.5× temple (20 : 8); in dorsal view, width of head 1.9× median length (62 : 33); POL : OD : OOL = 6 : 4 : 13 (Fig. 15); eye 3.4× temple (24 : 7); frons smooth, weakly concave.

***Mesosoma***. Length of mesosoma 1.6× its height (85 : 54); propleuron wide, deep and crenulate medially (Fig. 17); middle lobe of mesoscutum without impressions anteriorly; notauli deeper anteriorly, wider and flat posteriorly, with median longitudinal carina and transversely rugose posteriorly (Fig. 16); prescutellar sulcus narrow, crenulate; mesoscutal lobes sparsely punctate; mesopleuron rugose-punctate anteriorly, coriaceous medially, punctate ventrally (Fig. 17); metapleuron rugose-punctate; propodeum with deep crenulate depression, posterior V-shaped carina indistinct (Fig. 22); surface of propodeum rugose-punctate on anterior 0.7 of propodeum, almost smooth latero-posteriorly.

***Wings***. Length of fore wing 3.1× its maximum width (225 : 72); length of pterostigma 3.2× its width (48 : 15); fore wing vein SR1 5.1× as long as vein 3-SR (76 : 15); r : 3-SR : SR1 = 15 : 15 : 76; cu-a slightly reclivous (Fig. 18); cu-a : 2-CU1 = 12 : 34; 2-SR : 3-SR : r-m = 17 : 15 : 16; second submarginal cell of fore wing rather robust (Fig. 18); hind wing vein 1-M thick and evenly curved basally (Fig. 21); vein 1r-m of hind wing largely united with 1-SC+R (Fig. 21).

***Legs***. Hind coxa densely setose latero-ventrally, but without setae dorso-apically; length of femur, tibia and basitarsus of hind leg 4.0×, 9.4× and 6.7× their width, respectively; length of hind inner and outer tibial spurs 0.5× and 0.4× hind basitarsus, respectively; length of hind basitarsus 0.4× hind tibia (40 : 94) and 0.7× second-fifth tarsus (40 : 55); tarsal claw with large acute lobe (Fig. 19).

***Metasoma***. Length of first tergite 0.7× its apical width (31 : 45), with wide and deep basal excavation (Fig. 23); antero-lateral groove wide and deep, crenulate; median length of second tergite 0.85× third tergite (28 : 33); first metasomal tergite posteriorly, second-sixth metasomal tergites entirely coarsely reticulate; medio-apical tooth of sixth tergite developed; latero-apical groove of sixth metasomal tergite wide, crenulate (Fig. 24); length of ovipositor sheath 0.25× fore wing (18 : 72); ovipositor with dorsal nodus and apico-ventrally with serrations (Fig. 20).

***Colour***. Pale yellow; scapus dark brown; twenty four middle antennomeres yellow; palpi yellow; stemmaticum brown; mesoscutal lobes black, except median lobe posteriorly and lateral lobes anteriorly pale yellow; scutellum and metanotum, fore and middle legs pale yellow; hind leg dark brown to black, except coxa basally, trochantellus and lateral strip on hind femur yellow; hind tibial spurs pale yellow; propodeum largely black, pale yellow apically; pterostigma and veins brown; wing membrane subhyaline; large patches of six basal metasomal tergites black; ovipositor sheath brown; ovipositor reddish yellow.

#### Variations.

Length of body 5.4–8.4 mm, of fore wing 4.9–7.1 mm; antennae 65–70 antennomeres; 18–34 middle antennomeres yellow or antennae brown entirely; stemmaticum yellow; vein SR1 of fore wing 4.0–6.2× vein 3-SR; length of ovipositor sheath 0.22–0.3× fore wing; middle tarsus brownish yellow; hind tibial spurs brown.

**Male**. Unknown.

#### Biology.

Unknown.

#### Etymology.

The name of the species originates from the name of the country, where the holotype was collected.

#### Distribution.

N Vietnam: Son La, Thai Nguyen, Hoa Binh; S Vietnam: Pleicu.

#### Notes.

*Trispinaria vietnamica* sp. nov. differs from *T. seminigra* sp. nov. by having: first metasomal tergite with wide and deep basal excavation (narrow and deep in *T. seminigra*); median length of first metasomal tergite 0.7× as long as its apical width (0.9× in *T. seminigra*); propleuron wide and deep, crenulate (propleuron with distinct V-shaped carina posteriorly in *T. seminigra*); fore wing vein cu-a interstitial and weakly inclivous (vein cu-a vertical in *T. seminigra*); hind wing vein 1-M thick and curved basally (vein 1-M weakly curved basally in *T. seminigra*); middle coxa and mesopleuron pale yellow (black in *T. seminigra*); ovipositor apico-ventrally with serrations (apico-ventrally without serrations in *T. seminigra*).

The new species, *T. vietnamica* sp. nov., is similar to *T. maculata* van Achterberg, from India, Singapore, Sri Lanka, and Taiwan by sharing the following characters: vein 1r-m of hind wing nearly united with vein 1-SC+R; apical half of subbasal cell of fore wing largely glabrous; and frons smooth. The new species can be inserted into the key by van Achterberg (1991) as follows:

**Table:** 

7b.	Surroundings of stemmaticum of female yellowish brown; antenna near its apical 0.4 brown; face transversely rugose; fore wing vein r distinctly longer vein 3-SR (cf. fig. 42 in van Achterberg 1991); vein cu-a of fore wing vertical or nearly so, less inclivous than vein 3-CU1 (cf. figs 41 and 42 in van Achterberg 1991); length of ovipositor sheath 0.29–0.37× fore wing (fig. 42 in van Achterberg 1991). India, Singapore, Sri Lanka, Taiwan	***T. maculata* van Achterberg, 1991**
b’.	Surroundings of stemmaticum of female brownish yellow (Fig. 15); antenna dark brown basally and apically, near its apical 0.4 yellow; face punctate-coriaceous, except triangular area upper clypeus smooth; fore wing vein r as long as vein 3-SR (Fig. 18); vein cu-a of fore wing more or less inclivous than vein 3-CU1 (Fig. 18); length of ovipositor sheath 0.22–0.30× fore wing. Vietnam	***T. vietnamica* Long, sp. nov.**

**Figure 25.**
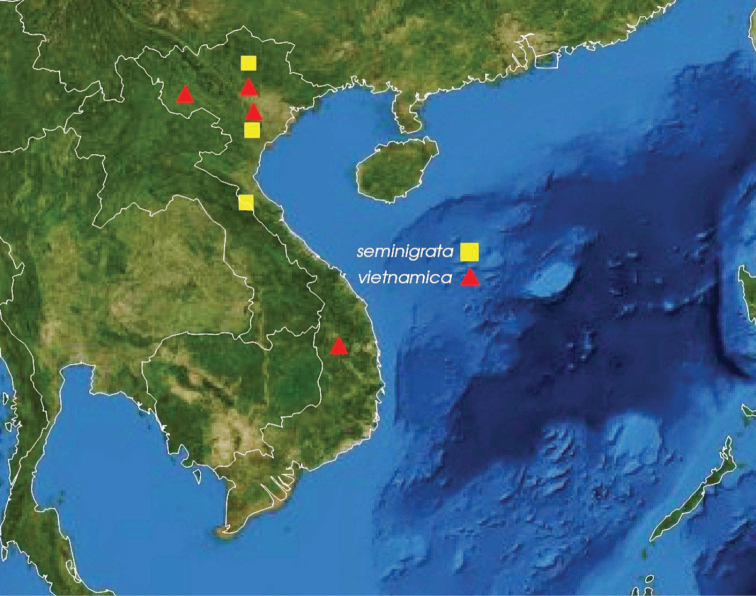
Distribution map of the two newly described species of *Trispinaria* in Vietnam.

## Discussion

The limitations in our paper are that the type specimens of nine species described by van Achterberg (1991) and one species by Wang et al. (2003) could not be examined. However, checking the original descriptions of all the known species revealed that two new species from Vietnam could be distinguished from the other *Trispinaria* species by their bicoloured antennae, except for two paratype specimens of *T. vietnamica* sp. nov. which have their antennae dark brown entirely. Comparisons of *Trispinaria* species from Vietnam show that they are distinguishable from two similar species from the Oriental region, i.e., *T. seminigra* sp. nov. vs. *T. sannio* (Enderlein, 1920) from Indonesia and Singapore; and *T. vietnamica* sp. nov. vs. *T. maculata* van Achterberg, 1991 from India, Singapore, Sri Lanka, and Taiwan.

The colour patterns of wasps seem to be one of the characters for distinguishing between *Trispinaria* species, including the two new ones from Vietnam. Apart from the bicoloured antennae, most specimens of *T. seminigra* sp. nov. that possess a black mesopleuron dorsally and metanotum were collected by using sweep nets in the forest understorey and by the Malaise traps set under the canopy forest in the northern and north central parts of Vietnam (Fig. 25). On the contrary, all the specimens of *T. vietnamica* sp. nov. were widely collected in the more open habitats, i.e., by Malaise trap(s) set in fruit orchards and by using sweep nets above bushes. The colour differences of the two new species discovered from Vietnam support van Achterberg’s argument, that in the tropical rain forests most of the large braconid wasps possess a dark(er) colour pattern than those from outside the forest (van Achterberg 1991).

## Supplementary Material

XML Treatment for Trispinaria

XML Treatment for Trispinaria seminigra

XML Treatment for Trispinaria vietnamica
